# Phα1β Spider Toxin Reverses Glial Structural Plasticity Upon Peripheral Inflammation

**DOI:** 10.3389/fncel.2019.00306

**Published:** 2019-07-10

**Authors:** Helia Tenza-Ferrer, Luiz Alexandre Viana Magno, Marco Aurélio Romano-Silva, Juliana Figueira da Silva, Marcus Vinicius Gomez

**Affiliations:** ^1^Centro de Tecnologia em Medicina Molecular, Universidade Federal de Minas Gerais (UFMG), Belo Horizonte, Brazil; ^2^Departamento de Saúde Mental, Faculdade de Medicina, Universidade Federal de Minas Gerais, Belo Horizonte, Brazil; ^3^Laboratório de Toxinas, Instituto de Ensino e Pesquisa da Santa Casa de Belo Horizonte, Belo Horizonte, Brazil

**Keywords:** glia, morphology, inflammatory pain, voltage-gated calcium channels, ω-MVIIA, Phα1β

## Abstract

The incoming signals from injured sensory neurons upon peripheral inflammation are processed in the dorsal horn of spinal cord, where glial cells accumulate and play a critical role in initiating allodynia (increased pain in response to light-touch). However, how painful stimuli in the periphery engage glial reactivity in the spinal cord remains unclear. Here, we found that a hind paw inflammation induced by CFA produces robust morphological changes in spinal astrocytes and microglia compatible with the reactive phenotype. Strikingly, we discovered that a single intrathecal injection with venom peptides that inhibit calcium channels reversed all the glial pathological features of the peripheral inflammation. These effects were more apparent in rats treated with the Phα1β spider toxin (non-specific calcium channel antagonist) than ω-MVIIA cone snail toxin (selective N-type calcium channel antagonist). These data reveal for the first time a venom peptide acting on glial structural remodeling *in vivo*. We, therefore, suggest that calcium-dependent plasticity is an essential trigger for glial cells to initiate reactivity, which may represent a new target for the antinociceptive effects of Phα1β and ω-MVIIA toxins in inflammatory pain conditions.

## Introduction

Sensory neurons from the dorsal root ganglia transduce nociceptive impulses from the periphery of the body to the spinal cord, which then relay the sensory information with local neurons and glial cells. Chronic pain states including tactile allodynia (in which sensitivity to innocuous stimuli is increased) are often present during a peripheral inflammatory event (Cervero and Laird, 1996; Samad et al., 2001). It is now clear that spinal glial cells in the dorsal horn engage with the establishment and maintenance of the inflammatory pain (Tappe et al., 2006; Milligan and Watkins, 2009; Sorge et al., 2015). Microglia and astrocytes enter a reactive state and form clusters in the vicinity of central terminals of injured sensory neurons (Sorge et al., 2015; Adams and Gallo, 2018), providing compelling evidence that glial cells remodel the maladaptive synaptic events in the nociceptive neurotransmission. For example, dysregulation of glutamate homeostasis via astrocytic uptake leads to central sensitization and persistent pain (Falnikar et al., 2016). Likewise, activated microglia mediate the mechanical hypersensitivity induced by colony-stimulating factor 1 (CSF1) and ATP derived from injured sensory neurons (Coull et al., 2005; Guan et al., 2015).

The notion that glial cells underlie the neurobiological mechanisms of nociception offers a new treatment approach for inflammatory pain. Notably, structural and gene expression maladaptive changes that are critical to initiate the reactive glial phenotype rely on calcium signaling (Westenbroek et al., 1998; Du et al., 1999; Bazargani and Attwell, 2016), presumably through activation of endogenously voltage-gated calcium channels (VGCC) (Latour et al., 2003). Upon activation, reactive glia depends on calcium mobilization to release proalgesic substances such as peptides, glutamate and proinflammatory mediators that contribute to the development of inflammatory pain (Bezzi et al., 1998; Bal-Price et al., 2002; McMahon et al., 2005; Tokuhara et al., 2010; Parkhurst et al., 2013; Lalo et al., 2014). Therefore, calcium signaling in astrocytes is an active modulator of neuronal activity (Di Castro et al., 2011; Panatier et al., 2011).

The pharmacological studies of peptide toxins ω-conotoxin MVIIA and Phα1β (purified from the venom of *Conus magus* and *Phoneutria nigriventer*, respectively) have progressed enormously in the last three decades. It is now clear that these toxins are useful analgesics (Wang et al., 2000; Staats et al., 2004; Souza et al., 2008; de Souza et al., 2011; Rigo et al., 2013; Diniz et al., 2014; Rosa et al., 2014). Both MVIIA and Phα1β appear to exert antinociceptive actions through inhibition of VGCC, which is expected to have an inhibitory role in pain transmission. The MVIIA toxin is a selective, reversible blocker of N-type VGCC (Kristipati et al., 1994; Knapp et al., 2012). The Phα1β toxin, otherwise, inhibits a variety of VGCC (including N-, R-, P/Q- and L-types) (Vieira et al., 2005) and the transient receptor potential ankyrin 1 (TRPA1) (Tonello et al., 2017). Surprisingly, despite the considerable advance from studies using models of chronic pain, it is still unclear whether the MVIIA and Phα1β toxins achieve analgesia as a result of the inhibition of glial reactivity in the spinal cord.

In the current study, we performed a morphological study to characterize the spinal glia response to peripheral inflammatory pain induced by complete Freund’s adjuvant (CFA). A single intrathecal injection of MVIIA and Phα1β produced a robust reversal of allodynia and glial plastic changes in a rodent model of peripheral inflammation. Our findings suggest that the antinociceptive effects of Phα1β and MVIIA in response to peripheral inflammatory injury may develop as a result of the calcium-dependent plasticity in glial cells. Thus, these data reveal for the first time a biological toxin that can modulate the structural remodeling of the glial morphology *in vivo*.

## Materials and Methods

### Ethics Statement

All animal care and experimental procedures were conducted in accordance with the ethical principles in animal experimentation (Zimmermann, 1983). The study protocol was reviewed and approved by the Ethics Committee in Animal Experimentation of the Federal University of Minas Gerais with the protocol number 347/2012.

### Animals

Male adult Wistar rats (180–250 g) were housed in a temperature-controlled facility with a 12 h light/dark schedule and provided with food and water *ad libitum*. The animals were habituated to the housing facilities for at least 1 week before the experiments began.

### Drugs and Treatments

The Phα1β toxin was purified from the venom of the spider *P. nigriventer* as previously described (Cordeiro Mdo et al., 1993). The ω-conotoxin MVIIA was purchased from Latoxan (Valence, France). Toxin stock solutions were prepared with phosphate buffer solution (PBS) to the required concentration on the day of the experiment. Freund’s Complete Adjuvant (CFA; Sigma-Aldrich, St. Louis, United States) consisted of 1 mg/mL heat-inactivated *Mycobacterium tuberculosis* in 85% paraffin oil and 15% mannan monooleate.

### Induction of Inflammatory Pain

Rats received a single subcutaneous intraplantar 50 μL injection of CFA or PBS (inflammation and control groups, respectively) into the right hind paw using a 27-gauge needle. Inflammatory pain was produced over a period of 2, 7, or 14 days (see the result section for details).

### Intrathecal Injection

The intrathecal injection was performed following the method previously described (Mestre et al., 1994). Briefly, on the CFA post-injection day 2, a single 10 μL intrathecal injection of Phα1β (200 pmol/injection), ω-conotoxin MVIIA (100 pmol/injection) or vehicle (PBS) was administered with a 28-gauge needle connected to a Hamilton microsyringe (Hamilton, NV, United States). During the procedure, the animals were lightly restrained to maintain the position of the needle. The puncture of the dura mater was indicated behaviorally by a slight flick of the tail.

### Von Frey Testing

Electronic Von Frey (EFF Insight, São Paulo, Brazil) was performed to measure the sensitivity to mechanical stimulation of the plantar surface of the hind paw. Rats were placed individually and habituated for 30 min into clear front Plexiglass boxes (9 × 7 × 11 cm) on an elevated mesh platform to allow access to the ventral surface of the hind paws. The tip of the pressure transducer of the analgesimeter was applied linearly through the holes in the mesh on the plantar surface of the hind paw at increasing pressure. Paw withdrawal caused by the stimulation was registered as a response and the corresponding force applied was recorded in grams to determine the mechanical sensitivity threshold. The average of five trials per paw was used to measure the mechanical algesia. Data were collected on both hind paws.

### Tissue Sections and Immunofluorescence

Rats were anesthetized with intraperitoneal ketamine/xylazine injection (100 mg/kg and 10 mg/kg, respectively) and transcardially perfused with ice-cold saline (0.9%) followed by freshly prepared 4% paraformaldehyde in PBS (pH 7.4). The spinal cords were removed by hydraulic extrusion with PBS and maintained in 4% PFA for 24 h at 4°C. Then, 50 μm transverse slices from the L3-L5 lumbar spinal cord were prepared using a vibrating blade microtome (Leica Microsystems; Wetzlar, Hesse, Germany). For BrdU detection, slices were pretreated for antigen-retrieval in 1M HCl for 30 min at 45°C and then washed with PBS. The free-floating slices were incubated with a permeabilizing-blocking solution (PBS containing 4% BSA and 0.2% Triton X-100) for 90 min at room temperature, and immunostained overnight at 4°C as previously described (Magno et al., 2019). We used the following primary antibodies at the indicated dilutions: anti-GFAP (Sigma-Aldrich, Cat# G3893, RRID:AB_477010, 1:400), anti-Iba1 (Wako, Cat# 019-19741, RRID:AB_839504, 1:400) and anti-BrdU (Abcam, Cat# ab1893, RRID:AB_302659, 1:500). Slices were rinsed in PBS and incubated for 3 h with the following Alexa Fluor (AF) dye-conjugated secondary antibodies: AF-594 anti-mouse (Thermo Fisher, Cat# A11005, RRID:AB_141372, 1:1,000), AF-488 anti-rabbit (Thermo Fisher, Cat# A21206, RRID:AB_2534073, 1:1,000) and AF-647 anti-sheep (Thermo Fisher, Cat# A-21448, RRID:AB_2535865, 1:1,000). For negative control, secondary antibody was incubated without the presence of the primary antibody. Following incubation, the tissue slices were washed again in PBS and mounted in Dako fluorescent mounting medium (Agilent Technologies, CA, United States). The region of interest (ROI) encompassing the laminae I-IV of Rexed in the dorsal horn was selected using the Leica Application Suite Advanced Fluorescence software (LAS-AF, Leica Microsystems; Wetzlar, Hesse, Germany). The most external border of the slice containing the fragments of the spinal meninges was not included in the quantification analyses (Choi, 1986). Confocal images (z-stacks) were acquired using a Leica SP5 microscope (Leica Microsystems; Wetzlar, Hesse, Germany). Acquisition settings were kept constant in each experiment for comparison between rats. All unsaturated images were captured under identical conditions (20× objective, resolution of 1,024 × 1,024 pixels and 200 Hz speed). Individual maximum z-projections were produced to calculate the fluorescent intensity of GFAP (LAS-AF software) or count manually Iba1-positive cells within the ROI using the LAS-AF software. For BrdU colocalization and microglia morphology experiments, images were acquired with a 63× objective. Colocalization experiments were performed in three confocal stacks images for each animal and evaluated using the orthogonal projection tool in Fiji/ImageJ software (Schindelin et al., 2012).

### Microglia Morphology

#### Skeletal Morphology

Z-stack images were acquired as explained above. Confocal images were then converted to binary images and edited manually to clear the background. Fiji Skeleton plugin (Arganda-Carreras et al., 2010) was applied to convert cells in skeletons, calculate the number and length of the ramifications, and the number of junctions.

#### Regularity Index (RI)

Binary images previously described were used to calculate the regularity index (RI) with the Fiji NND plugin (author: Yuxiong Mao), as described elsewhere (Davis et al., 2017). The RI was calculated as the ratio between *X_NND_/δ_NND_*, where *X_NND_* is the average nearest neighbor distance (NND) of the population and *δ_NND_* is the standard deviation NND of that population.

#### Roundness

Binary images previously described were modified to consider only the cell soma. The roundness was calculated using the Fiji analyze particles tool as described previously (Davis et al., 2017). The internal algorithm estimates the roundness (R) of the cells as *R = 4A/πM^2^*, where *A* is the area of the cell soma and *M* is the length of the major axis, driven from the longest axis of an ellipse fit to each cell soma. All morphological analyses were performed using Fiji/ImageJ (Schindelin et al., 2012).

### Luminex Assay

Pro- and anti-inflammatory protein levels in the spinal cord or dorsal root ganglia (DRG) samples were measured after 2 days of CFA injection. First, the L3-L5 spinal cord segment or DRG ipsilateral to the CFA-injected hind paw was dissected and stored at -80°C until analysis. Samples were homogenized in PBS with a protease inhibitor and a multiplexed immunoassay based on Luminex xMAP technology was performed for measuring IL-1β, TNF-α, IL-2, IL-4, IL-6, IL-10, IL-12p70, IL-13, IL-18, IFN-γ, GM-CSF, and VEGF (#RECYTMAG-65K, Millipore, Darmstadt, Germany). Assay plates were run according to the manufacturer’s protocol using a Luminex 200 equipment (Luminex, Austin, United States). Data were analyzed using the Luminex xPONENT^®^ software version 3.1. Total protein concentration for each sample was measured using the Pierce^TM^ BCA Protein Assay Kit (Thermo Fisher, MA, United States). Contents for each sample were displayed in pg/μL of mg of total protein.

### Glutamate Determination

Total glutamate determination was determined through a redox reaction and quantified in a spectrofluorophotometer (Shimadzu, RF-5301-PC). Tissue was homogenized in 0.1 mL of Krebs-Ringer-Hepes (KRH) buffer solution (116 mM NaCl, 4 mM KCl, 1 mM MgCl_2_.6H_2_O, 1.8 mM CaCl_2_.2H_2_O, 25 mM glucose and 10 mM HEPES, 7.4 pH). Then, 15 μL of the homogenized samples were added to a curvet containing KRH solution, 1 mM β-nicotinamide adenine dinucleotide phosphate (Sigma-Aldrich, #N6505-25MG) and 50 units of glutamate dehydrogenase (GDH, Sigma-Aldrich, #G2626). We quantified the levels of glutamate through the reduction of NADP^+^ to NADPH mediated by GDH. At the end of each quantification, 1 pmol glutamate was added to the reaction as a standard control. Delta was calculated for each step subtracting the final RFU from each stage by the initial RFU of the previous stage. Relative values of glutamate (nmol/μL) for each sample were normalized by total protein concentration using the Bradford Protein Assay (Sigma, #B6916-500mL).

### Statistics

For two-sample comparisons of a single variable, such as fluorescence intensity and cell counting of the CFA model, we used the unpaired Student *t*-test. One-way ANOVA followed by Tukey’s multiple comparison tests was performed to compare three groups or more with a single variable such as the effect of the toxin treatments on microglia morphology. For studying different groups with two variables, we used two-way ANOVA followed by Tukey’s multiple comparison tests (except otherwise stated, see the results). For the exact number of animals used in each experiment and details of statistical analyses, see the figure legend. All tests were two-tailed and had an alpha level of 0.05. All statistical analysis was performed using GraphPad Prism version 6 (GraphPad Software; La Jolla, CA, United States). Values were expressed as means ± SEM. Asterisks (^∗^) in the figures indicate the *P* values for the *post hoc* test and correspond to the following values: ^∗^*P* < 0.05; ^∗∗^*P* < 0.01; ^∗∗∗^*P* < 0.001, ^∗∗∗∗^*P* < 0.0001, based upon mean ± standard error of mean.

## Results

### Peripheral Inflammation Induces Astrocyte Reactivity in the Spinal Cord

**FIGURE 1:**
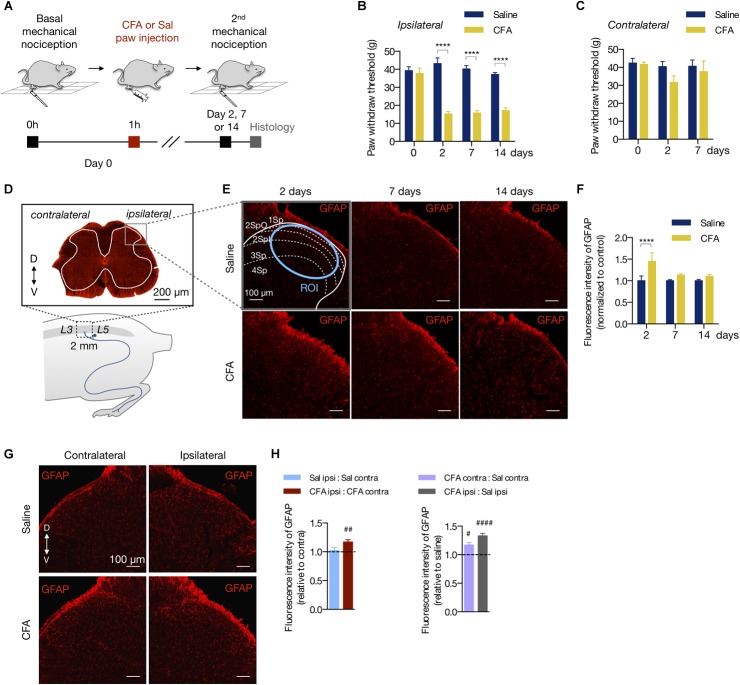
Peripheral inflammation induces astrocyte reactivity in the spinal cord. **(A)** Experimental design for the CFA (complete Freund’s adjuvant) model of peripheral inflammatory pain. One hour after the basal mechanical nociception, CFA or saline (Sal) were injected into the right hind paw of rats. Two, 7 or 14 days later, the second (2^nd^) mechanical nociception and histology were performed. **(B,C)** Mechanical nociception of the ipsilateral **(B)** and contralateral **(C)** hind paws was quantified before (day 0) and after saline or CFA injection (days 2, 7 and 14; *n* = 6/group). **(D)** Bottom: scheme showing the entry zone of the sciatic nerve in the lumbar spinal cord between L3 and L5 used for histology (∼ 2 mm). Top: GFAP-labeled astrocytes in a transverse section of the lumbar spinal cord (D = dorsal, V = ventral coordinates relative to the central canal). The ipsilateral and contralateral sides are relative to the paw injection. The gray matter is within the white line. The gray square represents the area of the dorsal horn acquired in the histology studies. **(E)** Representative fluorescent images and **(F)** quantification of GFAP-labeled astrocytes in the dorsal horn of the lumbar spinal cord, showing the saline and CFA groups in 2, 7, or 14 days after the paw injection. All images are from the ipsilateral side. The dashed and blue lines delimit the Rexed’s laminae and the region of interest (Savoie et al., 2019) used for the fluorescence intensity quantification, respectively. All values are relative to each respective saline group (*n* = 3 for saline and *n* = 4 for CFA groups, six slices per animal). **(G)** Representative fluorescent images of GFAP-labeled astrocytes in the dorsal horn of the spinal cord, showing the contralateral (left) and ipsilateral (right) sides of the Saline (Stevens et al., 2007) and CFA (bottom) groups. **(H)** Left: Intragroup analysis of GFAP fluorescence intensity of the ipsilateral relative to the contralateral side in each group (saline in blue, CFA in red). Right: Intergroup analysis of GFAP fluorescence intensity of the contralateral and ipsilateral sides of CFA-treated relative to the saline-injected rats (contralateral in purple, ipsilateral in gray; *n* = 4, six slices per animal; #*p* < 0.05; ##*p* < 0.01; ####*p* < 0.0001). In all figures, the scale bars represent 100 μm, except in figure **(D)** (200 μm). Summary data are represented as mean ± SEM. In figures **(B**,**C**,**F)**, ^∗∗∗∗^*p* < 0.0001.

Complete Freund’s adjuvant is widely used to study the mechanisms of persistent and peripheral pain (Raghavendra et al., 2004; Xu et al., 2010, 2014; Ikeda et al., 2012; Chen et al., 2015; Qi et al., 2016). To determine at which time point the CFA peripheral inflammation induces mechanical allodynia, we injected CFA or saline (0.9%) to the right hind paw of rats (50 μL per animal). Then, we tested the mechanical nociception 2, 7, or 14 days after the hind paw injection (Figure 1A). Compared to the saline group, CFA induced mechanical allodynia in the ipsilateral paw in all time points tested (2 days: 15.18 ± 1.45 g in CFA vs. 43.18 ± 3.12 g in saline; 7 days: 15.73 ± 1.32 g in CFA vs. 40.25 ± 1.84 g in saline; 14 days: 17.16 ± 1.43 g in CFA vs. 37.20 ± 1.00 g in saline; two-way ANOVA, treatment: *F*_(3,18)_ = 12.19, *p* = 0.001; time: *F*_(1,18)_ = 14.4, *p* < 0.001; interaction: *F*_(3,18)_ = 16.40, *p* < 0.001; Figure 1B). Conversely, the contralateral paw of the CFA group did not show a statistic reduction of the mechanical nociceptive threshold in any time investigated (2 days: 31.68 ± 3.65 g in CFA vs. 40.48 ± 2.78 g in saline; 7 days: 37.68 ± 5.86 g in CFA vs. 40.67 ± 3.42 g in saline; two-way ANOVA, treatment: *F*_(2,30)_ = 1.39, *p* = 0.264; time: *F*_(1,30)_ = 2.09, p = 0.159; interaction: *F*_(2,30)_ = 0.680, *p* = 0.514; Figure 1C).

To assess if the peripheral inflammation induces glial reactivity in the central nervous system (CNS), we measured the fluorescence intensity of the astrocyte marker GFAP (glial fibrillary acidic protein) in the dorsal horn of the lumbar spinal cord (L3 - L5) (Figure 1D). Peripheral inflammation, by CFA injection, increased the fluorescence intensity of GFAP in the ipsilateral dorsal horn 2 days after the CFA paw injection, compared to the saline group (Figure 1E,F). The increase of GFAP fluorescence intensity was not observed 7 or 14 days after inflammation onset (2 days: 44.9% ± 0.20; 7 days: 13.1% ± 0.03; 14 days: 10% ± 0.04, relative to the saline group; two-way ANOVA, treatment: *F*_(1,102)_ = 12.81, *p* < 0.001; time: *F*_(2,102)_ = 3.069, *p* = 0.051; interaction: *F*_(2,102)_ = 3.069, *p* = 0.051; Figure 1F). In the CFA group, GFAP fluorescence intensity was greater in the ipsilateral dorsal horn compared to the contralateral side, an occurrence that was not found in the saline group (intragroup analysis (ipsilateral vs. contralateral in the CFA and saline groups: 17% ± 0.04 (*p* = 0.002) and 2% ± 0.05 (*p* = 0.752), respectively; Student’s *t*-test; Figure 1G,H left). Interestingly, astrocyte reactivity was slightly higher in the contralateral side of the CFA group when compared to the contralateral side of the saline group (intergroup analysis, CFA:Sal ratio in ipsilateral: 33% ± 0.04 (*p* < 0.001); CFA:Sal ratio in contralateral: 17% ± 0.04 (*p* = 0.023); Student’s *t*-test; Figure 1H right).

Thus, the CFA-induced peripheral inflammation causes prolonged allodynia with acute effects on astrocyte reactivity in the dorsal horn of the spinal cord.

### Peripheral Inflammation Induces Microglia Reactivity in the Spinal Cord

**FIGURE 2:**
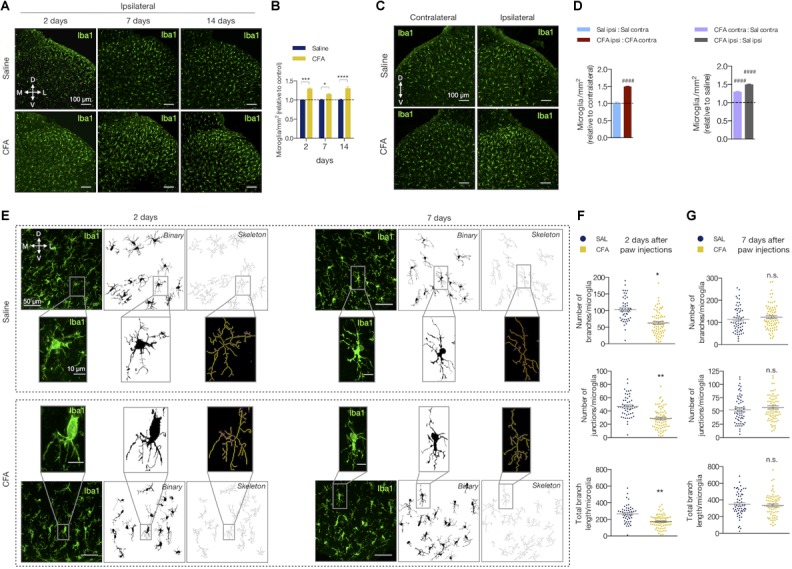
Peripheral inflammation induces microglia reactivity in the spinal cord. **(A)** Representative fluorescent images and **(B)** quantification of Iba1-labeled microglia (Iba1^+^) in the ipsilateral dorsal horn of the lumbar spinal cord, showing the saline and CFA groups in 2, 7, or 14 days after the paw injection. All values are relative to each respective saline group (*n* = 3 for saline and *n* = 4 for CFA groups, six slices per animal). **(C)** Representative fluorescent images of Iba1-labeled microglia in the dorsal horn of the spinal cord, showing the contralateral (left) and ipsilateral (right) sides of the saline (Stevens et al., 2007) and CFA (bottom) groups. **(D)** Left: Intragroup analysis of the number of Iba1^+^ cells of the ipsilateral relative to the contralateral side in each group (saline in blue, CFA in red). Right: Intergroup analysis of the number of Iba1^+^ cells of the contralateral and ipsilateral sides of CFA-treated relative to the saline-injected rats (contralateral in purple, ipsilateral in gray; *n* = 4, six slices per animal; #### *p* < 0.0001). **(E)** Representative images of the microglia morphology in the ipsilateral saline (Stevens et al., 2007) and CFA (bottom) groups in 2 (left) or 7 (right) days after the paw injection. Left panel: Iba1+ immunofluorescence; Middle panel: the binary transformation; Right panel: the resulting skeleton. The insets represent the respective higher magnification image. **(F**,**G)** Quantification of the microglia morphology parameters in ipsilateral side of 2 **(F)** or 7 **(G)** days after the paw injection. The graphs show the quantification of the number of branches (Stevens et al., 2007) and junctions (middle), or the total branches length (bottom) per microglia (*n* = 3–4 rats/group, three slices per animal). Each point in the graphs represents one microglia. Scale bars are 100 μm **(A,C)**, 50 μm **(E)** or 10 μm (**E**, insets). M: medial; L: lateral; D: dorsal; V: ventral (coordinates relative to the central canal). Summary data are represented as mean ± SEM. ^∗^*p* < 0.05; ^∗∗^*p* < 0.01; ^∗∗∗^*p* < 0.001; ^∗∗∗∗^*p* < 0.0001; n.s., not significant.

We also studied the effects of the peripheral inflammation on microglia reactivity in the spinal cord. The number of microglia (Iba1-labeled cells) increased after inflammation compared to the saline group in all time points tested (2 days: 29% ± 0.02; 7 days: 15% ± 0.02; 14 days: 30% ± 0.05, in CFA relative to the saline group; two-way ANOVA, treatment: *F*_(1,19)_ = 93.90, *p* < 0.001; time: *F*_(2,19)_ = 203.5, *p* < 0.001; *F*_(2,19)_ = 2.923, *p* = 0.078; Figure 2A,B). As for astrocytes, the CFA intragroup analysis 2 days after inflammation showed that the number of Iba1-positive cells was increased only in the ipsilateral dorsal horn compared to the contralateral side (ipsilateral vs. contralateral in the CFA and saline groups: 49% ± 0.02 (*p* < 0.001) and 0.5% ± 0.036 (*p* = 0.98), respectively; Student’s *t*-test; Figure 2C,D left). Similarly, intergroup analysis revealed that the number of microglia was higher in the contralateral side of the CFA group when compared to the contralateral side of the saline group (CFA:Sal ratio in ipsilateral vs. contralateral: 49% ± 0.02 (*p* < 0.001) and 29% ± 0.02 (*p* < 0.001), respectively; Student’s *t*-test; Figure 2D right).

When reactive, the microglia change their morphology and display different states of activation depending on the surrounding microenvironment (Kettenmann et al., 2011). Upon activation, microglia’s arborized appearance turns to a more phagocytic phenotype (including amoeboid morphology) with less ramifications, decreased process length and increased soma size. We then evaluated if peripheral inflammatory pain would also change the morphology of the spinal cord microglia (Figure 2E–G). The number of branches, junctions, and the total branch length per microglia were diminished after 2 days of CFA injection compared to the saline group (number of branches: 63.76 ± 8.02 (CFA) and 102.5 ± 3.57 (saline), mean difference (MD) = -38.70, 95% CI [-64.28 to -13.12], *t*_(6)_ = 3.89, *p* = 0.011, Student’s *t*-test; number of junctions: 29.08 ± 3.45 (CFA) and 46.70 ± 1.34 (saline), MD = -17.62, 95% CI [-28.52 to -6.73], *t*_(5)_= 4.16, *p* = 0.009, Student’s *t*-test; total branch length (μm): 175.5 ± 18.96 (CFA) and 268.9 ± 8.42 (saline); MD = -93.38, 95% CI [-143.1 to -43.65], *t*_(6)_ = 4.83, *p* = 0.005, Student’s *t*-test; Figure 2F). These morphological changes did not last long as these parameters were unaltered in the group analyzed 7 days after the inflammation onset (number of branches: 123.8 ± 7.43 (CFA) and 116.3 ± 9.31 (saline); MD = 7.46, 95% CI [-21.68 to 36.61], *t*_(6)_ = 0.627, *p* = 0.554, Student’s *t*-test; number of junctions: 29.08 ± 3.45 (CFA) and 46.70 ± 1.34 (saline); MD = -17.62, 95% CI [-28.52 to -6.73], *t*_(6)_ = 4.158, *p* = 0.009, Student’s *t*-test; total branch length (μm): 344.3 ± 16.72 (CFA) and 362.2 ± 18.98 (saline); MD = -17.95, 95% CI [-117.8 to 81.94] t_(6)_ = 0.440, *p* = 67.55, Student’s *t*-test; Figure 2G).

Therefore, the low-level peripheral inflammation with CFA induces microglial reactivity in the dorsal horn of the spinal cord.

### Peripheral Inflammation Did Not Alterthe Concentration of InflammatoryMediators and Glutamate in DRG norSpinal Cord

**FIGURE 3:**
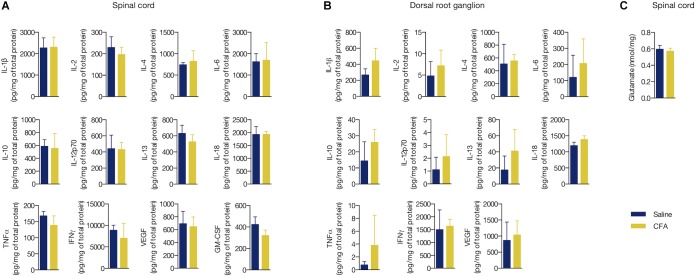
Peripheral inflammation did not alter the concentration of inflammatory mediators and glutamate in DRG nor spinal cord. **(A)** Cytokine and chemokine levels in the ipsilateral L3 - L5 spinal cord segment or **(B)** in the respective dorsal root ganglia (DRG) of the CFA- or saline-injected groups. All values are relative to the total protein content of each sample (mg/mL). GM-CSF was not detected in the DRG. **(C)** Glutamate levels in the ipsilateral L3 - L5 spinal cord segment. Summary data are represented as mean ± SEM (*n* = 3–4 rats/group).

Increased levels of cytokines and chemokines are known to signal inflammation (Turner et al., 2014). However, it is still unclear if a low-level peripheral inflammation is able to change the levels of these biomarkers in the CNS or sensory dorsal root ganglia (DRG). We performed a multiplex microbead assay for detection of 12 pro- and anti-inflammatory proteins (IL-1β, IL-2, IL-4, IL-6, IL-10, IL-12p70, IL-13, IL-18, TNF-α, IFNγ, VEGF, and GM-CSF) in the L3 - L5 segment of spinal cord and local DRG of CFA- or saline-treated rats for 2 days. We found no change in the tissue concentration of all studied biomarkers in these areas (CFA vs. saline, *p* > 0.05 in all cases, Student’s *t*-test; Figure 3A,B).

We also measured the levels of the excitatory amino acid glutamate, which has been consistently described to overexpress in response to inflammatory substances given peripherally (Shi et al., 2004; Souza et al., 2008; Vidal-Torres et al., 2012). Similarly to the pro- and anti-inflammatory biomarkers, no change in the glutamate levels in the spinal cord was found upon CFA treatment (glutamate (nmol/mg of protein): 0.57 ± 0.04 (CFA) and 0.596 ± 0.05 (saline), MD = -0.02, 95% CI [-0.17 to 0.12], *t*_(6)_ = 0.425, *p* = 0.685, Student’s *t*-test; Figure 3C). Conversely to glial reactivity, the low-level peripheral inflammation with CFA did not alter the concentration of selected pro- and anti-inflammatory biomarkers and glutamate in the spinal cord.

### The Calcium-Channel Blocker Toxin Phα1β Reverses Allodynia and Glial Reactivity

**FIGURE 4:**
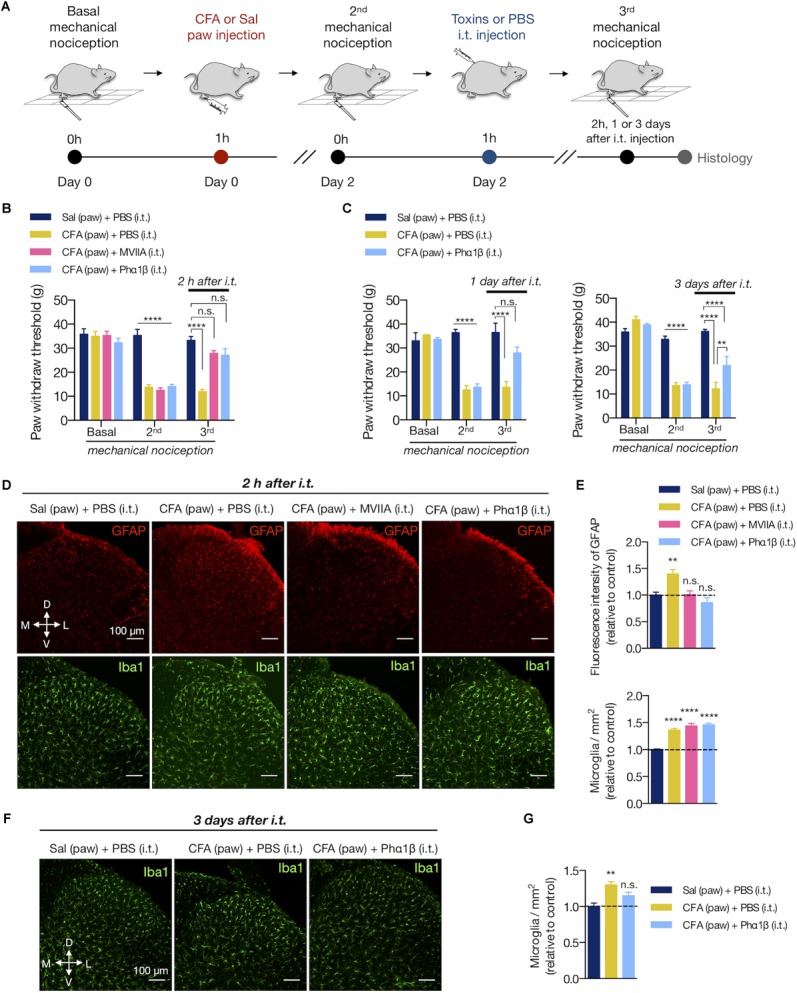
The calcium-channel blocker toxin Phα1β reverses allodynia and glial reactivity. **(A)** Experimental design for the CFA (complete Freund’s adjuvant) model of peripheral inflammatory pain followed by intrathecal (i.t) injections of PBS or calcium-channel blocker MVIIA and Phα1β toxins. After the basal mechanical nociception, CFA or saline was injected into the right paw (day 0). Two days later, the second (2nd) mechanical nociception was performed. Then, PBS or the MVIIA and Phα1β toxins were injected (i.t.). The third (3rd) mechanical nociception was performed 2 h, 1 or 3 days after the i.t. injection. Histology was carried out immediately after the 3^rd^ mechanical nociception. **(B)** Mechanical nociception of the ipsilateral hind paw was quantified before (Basal, day 0), after 2 days of saline or CFA paw injection (2nd, day 2) and 2 h after the i.t. injections (MVIIA, Phα1β or PBS, 3rd, day 2) (*n* = 7–8/group). **(C)** In an independent group of animals, the 3rd mechanical nociception of the ipsilateral hind paw was also quantified 1 day (left) or 3 days (right) after the i.t injection of Phα1β or PBS (*n* = 4/group). **(D)** Representative fluorescence images and **(E)** quantification of the fluorescence intensity of GFAP (Stevens et al., 2007) or the number of microglia (Iba1^+^, in the bottom) in the ipsilateral dorsal horn of the lumbar spinal cord 2 h after the i.t. injection (*n* = 3–4/group, six slices per animal). Graphs show values relative to the control group [Sal (paw) + PBS (i.t)]. **(F,G)** In an independent group of animals, the number of microglia (Iba1^+^) was also quantified 3 days after the i.t injection of Phα1β or PBS (*n* = 4/group, three slices per animal). The values in the graph are relative to the control group [Sal (paw) + PBS (i.t)]. Scale bars are 100 μm **(D,F)**. M: medial; L: lateral; D: dorsal; V: ventral (coordinates relative to the central canal). Summary data are represented as mean ± SEM. ^∗∗^*p* < 0.01; ^∗∗∗∗^*p* < 0.0001; n.s., not significant [compared to the Sal (paw) + PBS (i.t) group].

Animal toxins with analgesic properties have been considered as promising agents to treat persistent chronic pain (Staats et al., 2004; Rauck et al., 2006; Souza et al., 2008; de Souza et al., 2011; Rigo et al., 2013; Rosa et al., 2014; Turner et al., 2014; da Silva et al., 2015). The spider Phα1β and marine snail MVIIA toxins are inhibitors of VGCC, which are key molecules that transduce the nociceptive sensations through sensory neurons to CNS (Takahashi and Momiyama, 1993). However, as the VGCC are also important to trigger the reactive glial phenotype (Westenbroek et al., 1998; Du et al., 1999), we sought to investigate whether these therapeutic toxins would reverse the spinal glial response upon peripheral inflammation. We, therefore, injected intrathecally (i.t) PBS and these toxins into rats expressing the peripheral inflammatory pain 2 days after the CFA paw injection (Figure 4A,B). Phα1β and MVIIA toxins reversed the CFA-induced allodynia 2 h after intrathecal administration (3rd mechanical nociception (g) in CFA (paw) + PBS (i.t.) vs. CFA (paw) + MVIIA (i.t.) and CFA (paw)+ Phα1β (i.t.) groups: 11.93 ± 0.94 vs. 27.80 ± 1.25 and 27.07 ± 2.71, respectively; two-way ANOVA with Tukey’s multiple comparison test, treatment: *F*_(3,25)_ = 24.57, *p* < 0.001; time: *F*_(2,50)_ = 103.5, *p* < 0.001; interaction: *F*_(6,50)_ = 20.13, *p* < 0.001; Figure 4B).

We previously observed that Phα1β toxin treatment produced a robust, dose-dependent reversal of allodynia in rats for up to 24 h (de Souza et al., 2013). Here, we extended the analysis and observed that the analgesic effects of the Phα1β toxin could last longer, up to 3 days after a single intrathecal injection (3rd mechanical nociception (g) in CFA (paw) + PBS (i.t.) vs. CFA (paw)+ Phα1β (i.t.) groups: 12.20 ± 2.66 vs. 21.93 ± 3.82, respectively; two-way ANOVA with Tukey’s multiple comparison test, treatment: *F*_(2,9)_ = 26.59, *p* < 0.001; time: *F*_(2,18)_ = 107.9, *p* < 0.001; interaction: *F*_(4,18)_ = 25.65, *p* < 0.001; Figure 4C).

We next assessed if the Phα1β and MVIIA toxins reverse the CFA-induced glial reactivity (Figure 4D–G). After 2 days of the inflammation onset, both MVIIA and Phα1β toxins reversed the increases of GFAP fluorescence intensity 2 h after the i.t. injection (fluorescence intensity: 18.44 ± 0.35 [Sal (paw) + PBS (i.t.)], 24.82 ± 1.53 [CFA (paw) + PBS (i.t.)], 18.73 ± 0.86 [CFA (paw) + MVIIA (i.t.)], 16.86 ± 1.79 [CFA (paw) + Phα1β (i.t.)]; one-way ANOVA with Dunnett’s multiple comparison test, treatment: *F*_(3,37)_ = 6.438, *p* = 0.001; Figure 4D,E at the top). Conversely, the toxins did not reverse the CFA-induced number of microglia 2 h after treatment (microglia/mm^2^: 307.2 ± 11.06 [Sal (paw) + PBS (i.t.)], 456.5 ± 9.89 [CFA (paw) + PBS (i.t.)], 410.7 ± 8.41 [CFA (paw) + MVIIA (i.t.)], 447.0 ± 9.4 [CFA (paw) + Phα1β (i.t.)]; one-way ANOVA with Dunnett’s multiple comparison test, treatment: *F*_(3,83)_ = 48.83, *p* < 0.001; Figure 4E at the bottom). However, the Phα1β toxin diminished (∼50%) the CFA-induced number of microglia when the treatment effects were analyzed 3 days after the intrathecal injection (microglia/mm^2^: 509.6 ± 24.06 [Sal (paw) + PBS (i.t.)], 660.55 ± 23.81 [CFA (paw) + PBS (i.t.)], 585.35 ± 24.33 [CFA (paw) + Phα1β (i.t.)]; one-way ANOVA with Dunnett’s multiple comparison test, treatment: *F*_(2,9)_ = 9.829, *p* = 0.005; Figure 4F,G).

**FIGURE 5:**
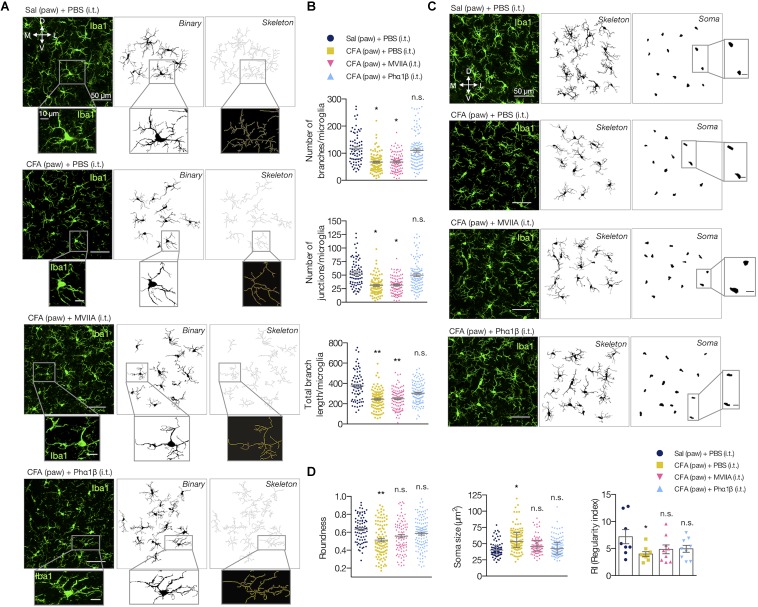
Phα1β reverses microglia morphology. **(A)** Representative images of the resulting ipsilateral skeleton and **(B)** branching complexity analyses 2 h after the i.t. injection (*n* = 3–4/group, three slices per animal). **(C)** Representative images show the resulting microglial soma appearance. The insets represent the respective higher magnification image. **(D)** Measurements for roundness (left), soma size (middle) and regularity index (RI, right). Each point in the graphs represents one microglia (except for RI, which depicts each animal). Scale bars are 50 μm **(A,C)** or 10 μm (insets). M: medial; L: lateral; D: dorsal; V: ventral (coordinates relative to the central canal). Summary data are represented as mean ± SEM. ^∗^*p* < 0.05; ^∗∗^*p* < 0.01; n.s., not significant [compared to the Sal (paw) + PBS (i.t) group].

We also evaluated if the Phα1β and MVIIA toxins could reverse the microglial morphological changes in the spinal cord upon CFA paw injection (Figure 5). Interestingly, the intrathecal administration of Phα1β, but not the MVIIA toxin, reversed the CFA-induced increases in the number of branches, junctions, and the total branch length per microglia (number of branches per microglia: 121.1 ± 11.39 [Sal (paw) + PBS (i.t.)], 61.49 ± 5.97 [CFA (paw) + PBS (i.t.)], 67.31 ± 9.87 [CFA (paw) + MVIIA (i.t.)], and 104.7 ± 22.05 [CFA (paw) + Phα1β (i.t.)], one-way ANOVA with uncorrected Fisher’s LSD test, treatment: *F*_(3,8)_ = 4.459, *p* = 0.0404); number of junctions per microglia: 55.24 ± 5.47 [Sal (paw) + PBS (i.t.)], 30.62 ± 2.90 [CFA (paw) + PBS (i.t.)], 32.24 ± 3.52 [CFA (paw) + MVIIA (i.t.)] and 51.71 ± 11.92 [CFA (paw) + Phα1β (i.t.)], one-way ANOVA with uncorrected Fisher’s LSD test, treatment: *F*_(3,8)_ = 3.413, *p* = 0.073; total branch length per microglia: 381.3 ± 23.66 [Sal (paw) + PBS (i.t.)], 243.5 ± 11.34 [CFA (paw) + PBS (i.t.)], 253.1 ± 18.31 [CFA (paw) + MVIIA (i.t.)] and 309 ± 22.21 [CFA (paw) + Phα1β (i.t.)], one-way ANOVA with Dunnett’s multiple comparison test, treatment: *F*_(3,8)_ = 10.59, *p* = 0.004; Figure 5A,B). These findings suggest that Phα1β toxin treatment attenuates the microglial activation upon peripheral inflammation.

We also studied the microglial somata morphology (Figure 5C,D). Peripheral inflammation ([CFA (paw) + PBS (i.t.)]) diminished the roundness of the soma when compared to the control group ([Sal (paw) + PBS (i.t)]). However, this typical morphological change was reversed with intrathecal administration of both Phα1β and MVIIA, as the microglial soma displayed a more roundness shape (0.632 ± 0.033 [Sal (paw) + PBS (i.t.)], 0.5120 ± 0.017 [CFA (paw) + PBS (i.t.)], 0.557 ± 0.017 [CFA (paw) + MVIIA (i.t.)] and 0.585 ± 0.007 [CFA (paw) + Phα1β (i.t.)]; one-way ANOVA with Dunnett’s multiple comparison test, treatment: *F*_(3,8)_ = 5.852, *p* = 0.0204; Figure 5D left). The peripheral inflammation also increased the microglial soma size. Likewise, both Phα1β and MVIIA reversed this morphological change as the microglial soma presented smaller size in the treatment groups (42.42 ± 3.95 [Sal (paw) + PBS (i.t.)], 56.58 ± 3.18 [CFA (paw) + PBS (i.t.)], 49.38 ± 3.63 [CFA (paw) + MVIIA (i.t.)] and 45.17 ± 1.57 [CFA (paw) + Phα1β (i.t.)]; Kruskal-Wallis with Dunn’s multiple comparison test, treatment: *F*_(4,12)_ = 6.90, *p* = 0.05; Figure 5D, in middle). We also studied the tissue distribution of microglia cells through evaluation of the regularity index (RI), a parameter that describes the regular spacing of cells. The inflammation reduced the RI, which was reversed with the Phα1β and MVIIA treatment (7.42 ± 1.03 [Sal (paw) + PBS (i.t.)], 3.98 ± 0.19 [CFA (paw) + PBS (i.t.)], 4.85 ± 0.86 [CFA (paw) + MVIIA (i.t.)] and 4.95 ± 0.81 [CFA (paw) + Phα1β (i.t.)]; one-way ANOVA with Dunnett’s multiple comparison test, treatment: *F*_(3,8)_ = 3.522, *p* = 0.069; Figure 5D right).

Altogether, these data suggest that the treatment with the analgesic calcium-channel blocker toxins Phα1β and MVIIA reverses the inflammatory pain and glial reactivity. Conversely to MVIIA, the Phα1β treatment reversed all the studied glial morphological changes induced by the peripheral inflammation.

### The Phα1β, but Not the MVIIA Toxin, Reverses Astrocyte Proliferation Induced by Peripheral Inflammation

**FIGURE 6:**
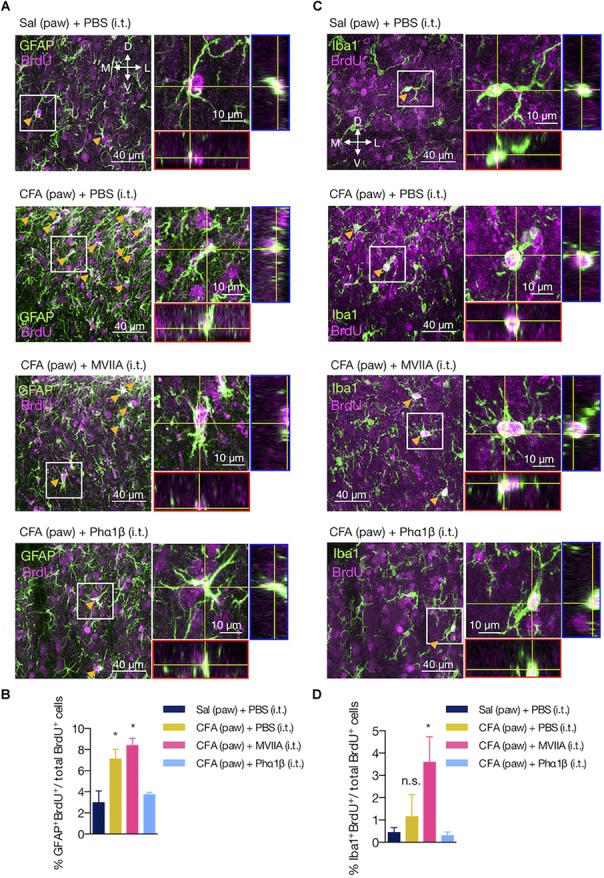
The Phα1β, but not the MVIIA toxin, reverses astrocyte proliferation induced by peripheral inflammation. **(A,C)** Representative images of **(A)** astrocyte and **(C)** microglia proliferation in the ipsilateral dorsal horn of the lumbar spinal cord. Arrows indicate examples of GFAP^+^BrdU^+^ or Iba1^+^BrdU^+^ cells. Insets contain the resulting confocal orthogonal view of the selected cells showing colocalization between GFAP or Iba1 with BrdU (in white). The lateral (blue) and bottom (red) rectangles represent the yz and xz views, respectively. **(B,D)** Quantification of **(B)** GFAP^+^BrdU^+^ or **(D)** Iba1^+^BrdU^+^ cells relative to the total number of BrdU^+^ cells (*n* = 3/group, three slices per animal). Scale bars are 40 or 10 μm for the low and high magnification images, respectively. M: medial; L: lateral; D: dorsal; V: ventral (coordinates relative to the central canal). Summary data are represented as mean ± SEM. ^∗^*p* < 0.05; n.s., not significant [compared to the Sal (paw) + PBS (i.t) group].

Glial cells often present the capacity to proliferate in the injured and uninjured CNS (Wanner et al., 2013; Bellver-Landete et al., 2019). To assess if the increases in GFAP intensity (astrocytes) and the number of Iba1-positive cells (microglia) were due to local proliferation, we determined the number of astrocytes and microglia cells that colabeled with the BrdU marker (Figure 6A–D). Peripheral inflammation increased the number of proliferating astrocytes (GFAP^+^BrdU^+^ cells), which was reversed by the Phα1β treatment, but not with the MVIIA administration (GFAP^+^BrdU^+^ relative to total BrdU^+^ cells: 2.98 ± 1.11 [Sal (paw) + PBS (i.t.)], 7.10 ± 0.92 [CFA (paw) + PBS (i.t.)], 7.89 ± 0.75 [CFA (paw) + MVIIA (i.t.)] and 3.74 ± 0.22 [CFA (paw) + Phα1β (i.t.)]; one-way ANOVA with Tukey’s multiple comparison test, treatment: *F*_(3,9)_ = 7.87, *p* = 0.007; Figure 6B). On the contrary, we did not observe local microglia proliferation induced by the CFA peripheral inflammation. Intriguingly, the treatment with the MVIIA toxin, otherwise, produced a significant increase in microglia proliferation (Iba1^+^BrdU^+^ cells) (Iba1^+^BrdU^+^ relative to total BrdU^+^ cells: 0.44 ± 0.22 [Sal (paw) + PBS (i.t.)], 1.15 ± 0.99 [CFA (paw) + PBS (i.t.)], 3.27 ± 0.91 [CFA (paw) + MVIIA (i.t.)] and 0.30 ± 0.16 [CFA (paw) + Phα1β (i.t.)]; one-way ANOVA with Tukey’s multiple comparison test, treatment: *F*_(3,9)_ = 2.94, *p* = 0.092; Figure 6D).

These findings suggest that astrocyte proliferation is a pathological hallmark of the peripheral inflammation, which can be reversed by the Phα1β toxin treatment.

## Discussion

Along with the critical role in maintaining the central nervous system (CNS) development and homeostasis, glial cells participate in the inflammatory response by becoming reactive after injury (Burda and Sofroniew, 2014). Whether peripheral inflammation shapes distinct glial responses in the damaged CNS remains a critical question to address. In this study, we demonstrated that a low-level peripheral inflammation triggers a complex and heterogeneous glial reactivity in the dorsal horn of the lumbar spinal cord. Notably, intrathecal administration of the Phα1β spider toxin, a VGCC blocker, rapidly reversed all the investigated features of glial reactivity upon peripheral inflammation. The findings of this study provide a new direction for understanding the antinociceptive effects of VGCC blocker toxins that may involve the structural remodeling of the glial morphology.

Proinflammatory molecules in the injury site sensitize the peripheric terminal of sensory neurons (Talbot et al., 2016), which then stimulate the central branch to release a number of proalgesic substances in the spinal cord, including substance P, excitatory amino acids and ATP. A number of studies showed that these substances released by the injured sensory neurons are capable to induce the reactive phenotype of resident glia surrounding these neurons within the spinal cord (Watkins et al., 2001a). For example, the release of colony-stimulating factor 1 (CSF1) induces microgliosis in the dorsal horn of the spinal cord (Guan et al., 2016). In response to injury, the reactive glia can adversely prolong the CNS inflammation and allodynia. Microglia elicit the first immune response to an insult in the CNS, typically within the first hours after injury, which is then followed by astrocytes (Jha and Morrison, 2018). Accumulating evidence suggests that microglia, once activated, can release inflammatory mediators that trigger astrocytes activation (Agulhon et al., 2012; Parpura et al., 2012). Interestingly, reactive astrocytes also release molecules that regulate the microglial response to inflammation. Therefore, although the functional boundaries between microgliosis and astrogliosis show different molecular pathways, the microglia-astroglia interaction upon neural damage is dynamic and very related to each other.

Here we observed that the peripheral inflammatory pain caused both astrogliosis and microgliosis in the ipsilateral dorsal horn of the lumbar spinal cord (L3-L5) 2 days after the CFA paw injection (Figure 1E, 2A). While astrogliosis was not observed after 7 days of the inflammation onset, the microgliosis persisted for up to 14 days. These findings indicate that the chronic microgliosis is a remarkable pathological feature in response to the CFA-induced inflammatory pain (Figure 1F, 2B). Since the microglial cells are known to reduce inhibitory synaptic inputs (Coull et al., 2005), the chronic microgliosis could mediate the inflammatory pain not only directly by strengthening the nociceptive synaptic connections, but also by physically displacing inhibitory terminals from surrounding reactive glia (Kambrun et al., 2018). Despite the fact that the CFA intragroup analysis showed increased gliosis in the ipsilateral dorsal horn relative to the contralateral side, CFA paw injection also caused a faint increase in gliosis within the contralateral side in comparison to the contralateral side of the saline group (Figure 1G,H, 2C,D). Possibly, the gliosis spread to the contralateral side might result from the intrinsic spinal commissural circuits (Petko and Antal, 2000; Petko et al., 2004), which discourages the use of the animal as its own control since the glial alterations did not appear to be strictly unilateral.

Reactive glia also alters their morphology in response to injury, which can offer valuable information about their state of activation (Fernández-Arjona et al., 2017; Heindl et al., 2018). Reactive microglia, for example, exhibit cytoskeletal alterations, including decreases in branching complexity (Jonas et al., 2012). Likewise, body size and processes rich in glial fibrillary acidic protein (GFAP) in astrocytes become thicker and highly immunoreactive (Sofroniew and Vinters, 2010; Pekny and Pekna, 2016). These changes in glia morphology are related to their functional state. For example, the morphological changes in the reactive glia promote the release of IL-1β, which amplifies the proinflammatory response (Fernández-Arjona et al., 2017). Accordingly, we observed that the peripheral inflammation induced morphological features of reactive microglia in the dorsal horn of the spinal cord. The number of branches, junctions, and the total branch length per microglia were diminished 2 days post the CFA injection compared to the saline group (Figure 2E,F). However, similar to astrogliosis, these morphological changes did not last long after the inflammation onset (Figure 2G). Since microglia promote activation of astrocytes, the changes of the microglial morphology to a basal state possibly resulted in the short-lasting astrogliosis, as described elsewhere (Raghavendra et al., 2003). These findings are in concordance with the fact that inflammation is a dynamic process involving a myriad of biological mechanisms, including microglial plastic changes (Morales et al., 2014). Possibly, the reactive microglial remodeling in the CNS upon peripheral inflammation manifests in the acute early proinflammatory response, which can be shortly reversed. This observation provides an opportunity to investigate if the microglial morphological transition to a less reactive phenotype could be critical for tissue recovery during the late anti-inflammatory responses.

Other studies have also shown that the pro-inflammatory peripheral stimuli are potent positive regulators of glial reactivity in the CNS. In most cases, however, this relationship was studied under experimental models with high levels of inflammation likely to disrupt the blood-brain barrier. Conversely to most reports describing paw injection volumes of CFA varying between 100 and 150 μL (Raghavendra et al., 2004; Lin et al., 2007; Xu et al., 2014), our rat model of peripheral inflammation received only 50 μL. Surprisingly, this low dose of CFA induced a significant and persistent mechanical allodynia that lasted up to 14 days (Figure 1B, 4B). However, the 12 inflammatory mediators studied after the CFA insult were unaltered in the lumbar spinal cord or dorsal root ganglia (Figure 3), which contrasts to previous studies (Samad et al., 2001; Raghavendra et al., 2004; Ledeboer et al., 2005). We cannot exclude the involvement of these inflammatory molecules in the pathological signs of the peripheral inflammation since our analysis was carried out 48h after the injury onset. Nevertheless, these data support that glial reactivity is potentially more sensitive to detect the CNS inflammatory effects of low doses of CFA.

We hypothesized that the overstimulated nociceptive endings located in the CFA injection site, possibly, provide a neuroimmune pathway mediating inflammatory signals toward the dorsal horn of the spinal cord, which could then induce spinal glial reactivity and strength of pain transmission (Watkins et al., 2001b; Woolf and Ma, 2007). It is well known that the release of pro-nociceptive neurotransmitters by sensory afference into the superficial laminae of the dorsal horn depends on the calcium influx through presynaptic terminals (Augustine, 2001). Therefore, this previous evidence might explain the suppressor effects of VGCC blockers such as the spider toxin Phα1β on abnormal sensory firing resulting in analgesia (Figure 4B,C). Although both Phα1β and MVIIA reduced the astrocytic reactivity with only 2 h of treatment, none of them could repair the increased number of microglia induced by CFA in this period. In contrast to MVIIA (de Souza et al., 2013), the Phα1β treatment provides a more durable analgesic benefit that persists for up to 3 days (Figure 4C). Therefore, we investigated the long-term effects only for the Phα1β toxin. Surprisingly, Phα1β decreased the number of microglia in CFA-treated rats 3 days after a single intrathecal injection (Figure 4F,G). Interestingly, whereas the Phα1β toxin failed to change the increased number of microglia 2 h after injection, it reversed all the studied morphological features of reactive microglia caused by inflammation (Figure 5A–D). Phα1β toxin also reversed other morphological changes induced by CFA such as the decreased distance between the glial bodies (regularity index) and distribution homogeneity (Pomilio et al., 2016; Davis et al., 2017). This observation is unprecedented and raises the question of whether the Phα1β toxin, apart from its traditional use as an antinociceptive agent, may be a promising anti-inflammatory drug in the CNS. It should also be noted that the treatment with the toxin MVIIA presented a less effective reversal of the morphological changes associated with CFA administration, which were restricted to the microglial soma (roundness and soma size).

Spinal cord gliosis following peripheral inflammation might result from either migration toward the dorsal horn or local proliferation (by producing newly proliferated BrdU^+^ glia) (Suter et al., 2007). We observed that CFA paw injection increased the number of proliferated astrocytes (GFAP^+^BrdU^+^) in the lumbar dorsal horn (Figure 6A,B), indicating that increases in GFAP fluorescence intensity result from both proliferation and astrocytic hypertrophy (Figure 1). Conversely, peripheral inflammation did not alter the number of newly proliferated microglia (Iba1^+^BrdU^+^) (Figure 6C,D). Taking together, these findings suggest that migrating microglia (and/or infiltrating macrophages) from other regions of the spinal cord contribute to dorsal horn microgliosis and support the notion that heterogeneous glial populations respond differently to the peripheral inflammation.

The phenotype changes in the spinal glia depend on calcium signaling. The glia display a variety of calcium channels that, along with the intracellular Ca^2+^ storages, mediate the gene expression and trigger the release of neuroactive substances in response to a variety of stimuli, including inflammation (Verkhratsky et al., 1998). Therefore, the temporal and spatial oscillations in the intracellular calcium levels are important for the glia plasticity (Eichhoff et al., 2011), especially when located within the peripheral processes (Lee et al., 2010). Specifically, both microglia and astrocytes express a wide array of different VGCC types that are key mediators of calcium entry from the extracellular space, thereby regulating the glial autocrine release, structural remodeling, and proliferation (Sontheimer, 1994; Verkhratsky and Kettenmann, 1996; Stebbing et al., 2015). In corroboration with recent evidence (Zamponi, 2016), we also proposed these calcium channels as therapeutic targets in CNS inflammation because the glial calcium signaling may provide positive feedback to the establishment of the reactive phenotype (Westenbroek et al., 1998; Du et al., 1999). In contrast to the MVIIA toxin, we observed that Phα1β reversed the increased number of newly proliferated astrocytes following the peripheral inflammation (Figure 6A,B). The MVIIA toxin is a peptide isolated from the venom of the *C. magus* snail, regarded as a highly selective blocker of N-type Ca^2+^ channels (Olivera et al., 1987; Kristipati et al., 1994). The Phα1β, on the other hand, has been shown to block different VGCC subtypes including N, R, P/Q and L-types (Vieira et al., 2005), in some cases even the TRPA1 channel (Tonello et al., 2017). Possibly, the remarkable effect of the Phα1β on the astrocytic proliferation could thus be critically dependent on the blockage of the VGCC other than the N-type.

## Conclusion

In conclusion, our findings demonstrate for the first time that analgesic VGCC blocker toxins remodel the glial morphology upon peripheral inflammation. Whether this effect arises from the direct inhibition of VGCC in glia or sensory neurons is yet to be determined. We, therefore, suggest that the Phα1β spider toxin, apart from its analgesic effects, is also a potent anti-inflammatory compound acting on glial reactive phenotype.

## Data Availability

The datasets generated for this study are available on request to the corresponding author.

## Ethics Statement

The experiments were carried out following Ethical guidelines for investigations of experiments in conscious animals (Zimmermann, 1983) and were authorized by the Universidade Federal de Minas Gerais (Protocol No. 347/2012).

## Author Contributions

All authors conceived and designed the experiments. HT-F and JdS conducted the drug treatments and behavioral tests. HT-F and LM carried out histology and morphological analysis. MR-S and MG contributed with ideas and discussions. HT-F and LM wrote the manuscript.

## Conflict of Interest Statement

The authors declare that the research was conducted in the absence of any commercial or financial relationships that could be construed as a potential conflict of interest.
